# PP13 and PAPP-A in the First and Second Trimesters: Predictive Factors for Preeclampsia?

**DOI:** 10.5402/2012/263871

**Published:** 2012-06-18

**Authors:** Narges Moslemi Zadeh, Farshad Naghshvar, Sepideh Peyvandi, Parand Gheshlaghi, Sara Ehetshami

**Affiliations:** ^1^OB/GYN Department, Imam Khomeini Hospital, Mazandaran University of Medical Sciences, Sari, Iran; ^2^Pathology Department, Imam Khomeini Hospital, Mazandaran University of Medical Sciences, Sari, Iran; ^3^Education Development Center (EDC), Mazandaran University of Medical Sciences, Sari, Iran

## Abstract

*Background*. Preeclampsia affects 5-6% of all pregnancies. Predictive factors of preeclampsia can be helpful in early diagnosis of this disease. In this study the predictive values of biochemical markers placenta protein 13 (PP13) and pregnancy-associated plasma protein A (PAPP-A) have been assessed in early diagnosis of preeclampsia. *Methods*. This case-control study was conducted on 1500 women who presented to a healthcare center of Sari, Iran, between 2010 and 2011. Blood samples were drawn in weeks 11–13 and 24–28 of pregnancy. Of them who developed preeclampsia were considered as case group. A control group consisted of similar women regarding mean age, body mass index (BMI), and pregnancy age. PAPP-A and PP13 serum levels were measured. Data were analyzed using proper statistical tests. *Results*. PAPP-A and PP13 serum levels were significantly lower in both the first and second trimesters in women who developed preeclampsia (*P* < 0.001). The cumulative value of all four variables with cut-off point of 238.5 has sensitivity, specificity of 91.0%, and undercurve surface of 0.968 which is the most diagnostic value for preeclampsia. *Conclusion*. It is possible to advantage measuring of PAPP-A and PP13 in the first and second trimesters especially their cumulative values in both trimesters for prediction of the incidence of preeclampsia.

## 1. Introduction

Preeclampsia is defined as systolic blood pressure equal to or more than 140 mmHg and diastolic blood pressure equal to or more than 90 mmHg associated with proteinuria equal to or more than 300 mg in 24 hr urine or 1+ result in dip-stick test which occurs after weeks 20 of pregnancy [1]. Preeclampsia affects 5-6% of all pregnancies and accounts for 18% of maternal deaths during pregnancy and accompanied with bleeding and infection is one of the three leading causes of death among pregnant women worldwide [2]. Placenta protein 13 (PP13) is a dimer 32 k dalton protein which is produced by placenta and has role in implementation. Recently this protein has been attracted as a probable marker for early diagnosis of preeclampsia [3, 4]. During normal pregnancy, the PP13 serum levels increase while the declined levels have been found in patients who developed preeclampsia. Some studies have shown that measuring PP13 levels in the first trimester has predictive value.

In the study of Nicloides et al. in patients with pregnancy termination due to severe preeclampsia before week 34, the PP13 serum levels were lower than the normotensive individuals [5]. A retrospective study showed that PP13 levels in weeks 5 and 6 of pregnancy are associated with incidence of preeclampsia [6].

PAPPA-A is produced by growing trophoblast and indirectly induces aggression to the endometrium and increases during pregnancy [7].

PAPP-A is also a relative predictive factor of preeclampsia, but its predictive value is not as precise as PP13 and Doppler sonography [8]. Decreased levels of PAPP-A are probably associated with incidence of preeclampsia. Several studies have been illustrated that there is association between decreased PAPP-A levels in the first trimester and incidence of early preeclampsia [3, 9]. The decreased PAPP-A levels, are seen in all trimesters in women with preeclampsia [10]. It is not approved whether early evaluation of risk factors using PP13 and PAPP-A, alone or in combination, can improve pregnancy outcomes or not [1]. The predictive values of markers which show function of placenta like PP13 and PAPP-A in the second trimester have not been clearly defined and there is controversy over studies [11]. considering the importance of preeclampsia and its severe consequences like maternal and fetal death, and the probable positive role of chemical biomarkers such as PAPP-A and PP13, the study was aimed to evaluate predictive value of these two markers alone or in combination with each other in the first and second trimesters as cumulative value in prediction of preeclampsia.

## 2. Materials and Methods

This prospective nested case-control study was conducted on 1500 women who were presented for prenatal care to health-treatment center of Sari, Iran, between 2010 and 2011. Inclusion criteria were normotensive singleton pregnant women who were presented in their first trimester. Exclusion criteria were history of cardiovascular diseases, chronic hypertension, smoking, and diabetes. Demographic characteristics including mother's age, weight, body mass index, age of pregnancy, and hypertension were gathered in a prepared questionnaire. The study was approved by ethical Committee of Mazandaran University of Medical Sciences. The sample size was calculated considering 95% confidence interval (CI) and power of 80%; each case and control group consists of 100 patients. Subsequent completing questionnaire and giving informed consent, 10 cc blood sample was drawn in weeks 11–13 as a sample of the first trimester. Specimens were centrifuged (4000/min) in 10 min and held on −70°C. Then, in weeks 24–28 participants again were referred and a similar blood sample was taken and held in similar situation. These pregnant women were followed up to labor time. At labor time, all participants were assessed and those with preeclampsia were defined and classified in case group (100). A control group, consisted of women with normal pregnancy and similar age, pregnancy age, and BMI to the case group, was recruited. Patients data including types of delivery, pregnancy age at the time of pregnancy elimination, preeclampsia severity, and infant's gender and its weight were recorded. Preeclampsia group was divided into two groups: early and late preeclampsia. In all studied participants, serum PAPP-A (IBL kit, Germany) and PP13 (Life science, China) levels in the first and second trimesters were measured using ELISA by technicians who were not aware of pregnancy outcomes. Serum PAPP-A and PP13 levels in each group and between groups were compared using students *t*-test and chi-square and ANOVA tests using SPSS 16.0. Diagnostic values of these markers were defined using specificity and sensitivity. Cut-off points were defined using Roc curve.

## 3. Results

Of 1500 pregnant women who were followed in this study, finally 100 women with preeclampsia were classified in case group and 100 other women with normal pregnancy with matched BMI, age, and pregnancy age were recruited as control group. Demographic characteristics in both case and control groups have been shown in Table 1. As it is mentioned in the table, mother's age, weight, types of delivery, and infant's birth weight did not differ significantly between two groups.

The mean serum PAPP and PP13 levels were significantly lower in the first and second trimesters in preeclampsia group compared to control group.

**Figure 1:**
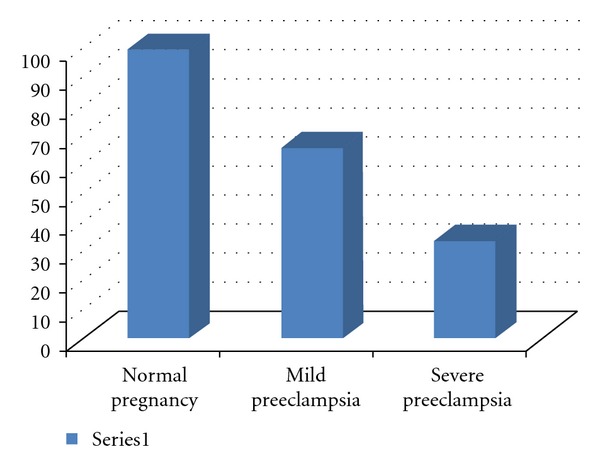
Frequency of mild and severe preeclampsia.

Of 100 women with preeclampsia 66 cases have mild preeclampsia while 34 cases have severe preeclampsia (Figure 1), the mean serum levels of measured markers in the first and second trimesters significantly differ between these two groups. Distributions of markers in each group have been summarized in Table 2.

The mean serum PAPPA and PP13 levels in the first trimester were significantly lower in women with early preeclampsia (before week 34) compared to late preeclampsia (after week 34) (*P* < 0.005 and *P* < 0.04, resp.); but this difference was not significant in the second trimester (Table 3).

Using Roc curve the cut-off point of PAPPA in the first and second trimesters were calculated as 1.65 and 100.5.  Table 4 shows the specificity and sensitivity of this marker in this cut point. The cut-off points of PP13 in the first and second trimesters were 88.5 and 315.5, and its sensitivity and specificity are mentioned in Table 4.

Based on the results of Tables 5 and 6, three different linear combinations of studied markers were created. In the first two situations, linear combinations of two markers for each time session, and in the third situation, values of two markers in two time sessions (in sum four variables) were inserted in the linear combination. Then using analysis of Roc curve, surface undercurve, and cut-off points for these three linear combinations and various factors of validity like specificity, sensitivity, and positive and negative predictive values were estimated. Results are summarized in Table 7.

Thus, comparing two linear combinations in the first and second trimesters, it seems that using both markers for prediction of preeclampsia in pregnant women in the first and second trimesters is associated with more validity compared to each marker alone. Meanwhile, if it is possible to measure both markers in both time sessions, using a four-variable linear combination will help to better prediction and diagnosis of preeclampsia. In the first trimester it is necessary just to sum PAPPA value with 0.1 of PP13 and values ≤10.9 would be positive. In the second trimester, it is necessary to sum PAPPA values with 1/3 of PP13 values and values ≤214.5 would be positive. For calculation of four-variable combination, it is necessary to sum 10-fold of PAPPA with PP13 in the first trimester, and 1/2 of PAPPA value with 1/4 of PP13 value in the second trimester; values ≤238.5 would be positive. In the other approach multiple diagnostic tests were assessed to evaluate this hypothesis that both diagnostic preeclampsia markers would be more helpful in the prediction than each marker alone. In this approach parallel tests were applied. It means that it was assumed that two tests are performed simultaneously. In parallel tests it is possible to consider two different situations: in the first one, when both two tests are positive the patient is considered positive and in the second situation only one positive test is considered as positive. Calculated cut-off points were used in these analyses and various validity factors like sensitivity, specificity, and positive and negative predictive values were estimated. Results are summarized in Table 8.

## 4. Discussion

In this study we showed that the pregnancy-associated plasma protein-A (PAPP-A) has been lower in both first and second trimesters among women who developed preeclampsia (*P* < 0.001). Meanwhile, the decreased levels of this marker associated with incidence of early preeclampsia in the first trimester (*P* = 0.05), but in the second trimester there was not a significant difference among patients with early and late preeclampsia (*P* > 0.05). Its serum levels in the first and second trimesters were significantly lower among those with severe preeclampsia compared to mild preeclampsia (*P* < 0.001). Its cut-off point in the first trimester was defined 16.5 which has sensitivity, specificity, and positive and negative predictive value of 60.0%, 78.0%, 73.2%, and 66.1%, respectively. The cut-off point of PAPP-A in the second trimester was 10.5 which has a sensitivity and specificity of 71.0%, and positive and negative predictive value of 71.0%.

In a study by Poon et al. [12] the PAPP-A serum levels in the first trimester was under fifth percentage in 21.9% and 6.5% of patients with early and late preeclampsia. In a study by F. Audibert et al. [13], considering 10% of false positive, the sensitivity of PAPP-A serum levels was 35.2% and 55.6% in the diagnosis of preeclampsia and early preeclampsia.

N. A. Bersinger et al. [14] also showed that PAPP-A levels were significantly lower among those women who developed preeclampsia in the first trimester. Indeed, it can be concluded that declined PAPP-A levels may be a predictive marker for existence of a basis for incidence of preeclampsia especially early preeclampsia.

In the study by F. Audibert et al. in 2010, it has been found that combination of mother's clinical characteristics and serum biomarkers (PIGF, Inhibin-A, and PAPP-A) in the first trimester was found in 75% of cases with early preeclampsia, but addition of uterine artery Doppler, PP13 and metalloproteinase 12 did not improve diagnosis of early preeclampsia [13].

In our study, the serum levels of placenta protein 13 (PP13) were found lower in both first and second trimester among women who developed preeclampsia compared to the control group (*P* < 0.001). Meanwhile, PP13 levels were significantly lower in the first trimester among women with early preeclampsia compared to late preeclampsia (*P* = 0.047), but its levels did not differ in the second trimester (*P* > 0.05). Its serum levels in both first and second trimesters were significantly lower in women with severe preeclampsia compared to mild preeclampsia (*P* < 0.001). The sensitivity, specificity, and positive and negative predictive values of this marker with cut-off point of 88.5 in the first trimester were 77.0%, 82.0%, 81.1%, and 78.1%, respectively. In the second trimester the cut-off point was 315.5 and its sensitivity, specificity, and positive and negative predictive values were 79.0%, 80.0%, 79.8%, and 79.2%, respectively.

A new variable was conducted using modeling and linear equation of PP13 and PAPP-A in the first (PAPP-A+ (PP13*0.1) and second (PAPP-A+ (PP13*2/3) trimesters. The cut-off point of this new variable in the first trimester was 10.9 and its sensitivity, specificity, and positive and negative predictive values were 86.0%, 80.0%, 81.1%, and 85.1%, respectively. Meanwhile, the cut-off point of this new variable in the second trimester was 214.% which had sensitivity, specificity, and positive and negative predictive values of 89.0%, 81.0%, 82.4%, and 88.0%, respectively. Therefore, the new variable resulted from linear equation of PP13 and PAPP-A had higher sensitivity and specificity compared to each variable alone and using this equation would be more helpful in early diagnosis of preeclampsia. The cumulative value of all four variables with cut-off point of 238.5 had sensitivity and specificity of 91.0% and undercurve surface of 0.968 which is the most diagnostic value for preeclampsia.

Parallel test was employed to assess the usefulness of simultaneous interpretation of both tests. The specificity was calculated 96.0% and 97.05 in the first and second trimesters in the cases of being positive of both tests; this would be so valuable to rule out of suspicious cases.

In a study by Ghafets et al. [3] PP13 serum levels in the first trimester had a sensitivity of 79.0% and 28.0% in patients with preeclampsia (All cases) and early preeclampsia considering specificity of 90.0%. in another study by Spencer et al. [9], considering the specificity of 80.0%, PP13 has a sensitivity of 50% in diagnosis of early preeclampsia and 44% in diagnosis of all cases of preeclampsia, but in this study adding PAPP-A to PP13 did not improve its sensitivity and specificity and did not have any effect in better diagnosis of preeclampsia. Adding PP13 to placental vessels Doppler sonography in the first trimester improved the sensitivity and specificity of Doppler sonography in prediction of preeclampsia. In contrary to other studies, F. Audibert et al. [13] mentioned that the accuracy of PP13 is week for prediction of preeclampsia. Nikolaides et al. [5] reported the sensitivity of PP13 80.0% considering the false positivity of 10.0%, while they found the sensitivity of placental Doppler sonography 40.0%. Meanwhile, they reported that adding PP13 to Doppler sonography in the first trimester increased its diagnostic sensitivity to 90.0%. It is important to mention that none of previous studies have employed linear equation of PAPP-A and PP13. Odibo et al. in a study in 2011 on PAPP-A, PP13, and uterine artery index in 42 cases of preeclampsia in the first trimester and they presented in 49.0%, 58.0%, and 62.0% of women with preeclampsia. PP13 was the best predictive marker in the first trimester with the sensitivity of 79.0%. They found that PAPP-A, PP13, and uterine artery index are each one a predictive marker of preeclampsia in the first trimester, but their combination did not increase the diagnostic value of preeclampsia [15].

Infants' weight did not differ significantly between our two studied groups (*P* = 0.136), while in studies by Khalil [16] and Ghafetz [3] mothers with preeclampsia had babies with lower weight and also were much underwent cesarean section. In our study, types of delivery (vaginal/cesarean) did not differ significantly between two groups with normal pregnancy and preeclampsia (*P* = 0.57).

The effects of PP13 on implementation and remodeling of mother-site placental vessels is not completely defined, but it is known that PP13 is produced in placenta is banded to carbohydrate particles in extracellular matrix which have role in placental implementation. Moreover, PP13 increases releasing of prostaglandins which improve vascular remodeling and placental development [13, 17]. Thus, it is possible that its decreased levels interfere with functions which are necessary for placental development, and so decline in its levels in the first trimester can be a predictive marker for incidence of preeclampsia to follow.

Thus, given our findings, it is possible to benefit measuring of PAPP-A and PP13 in the first and second trimesters especially their cumulative values in both trimesters for prediction of incidence of preeclampsia with high specificity and sensitivity. The resulted linear equation increases diagnostic specificity and sensitivity and make it possible to advantage appropriate preventive and treatment strategies by better prediction of preeclampsia.

## Figures and Tables

**Table 1:** Distribution of pregnant women with preeclampsia and control based on systolic and diastolic blood pressures, mother's weight and age, infant's weight, and types of delivery.

Variables	Control	Preeclampsia	*P* value
Mother's age	26.5 ± 5	25.9 ± 4.9	0.712
Mother's weight	79 ± 8	79 ± 8.7	0.946
Systolic blood pressure	114 ± 4.9	147 ± 9.8	0.0001
Diastolic blood pressure	74 ± 4.9	94 ± 10.4	0.000
Infant's birth weight	3170 ± 328	3250 ± 400	0.134
Vaginal delivery	57%	52%	0.57
Cesarean section	43%	48%	0.57

**Table 2:** Distribution of studied markers in each group in the first and second trimesters.

Markers	Control	Mild preeclampsia	Severe preeclampsia	*F* factor	*P* value
PAPPA first trimester	2.7 ± 1.4	1.7 ± 0.4	0.99 ± 0.4	38.0	<0.001
PP13 first trimester	101.7 ± 16.8	80.5 ± 14.4	54.5 ± 18.9	111.9	<0.001
PAPPA second trimester	113.2 ± 14.3	97.2 ± 16.8	90.0 ± 12.8	38.8	<0.001
PP13 second trimester	377.9 ± 69.5	288.6 ± 55.4	244.8 ± 57.4	75.6	<0.001

**Table 3:** Distribution of pregnant women with early and late preeclampsia based on the PAPPA and PP13 in the first and second trimesters.

Preeclampsia	PAPPA first trimester	PAPPA second trimester	PP13 first trimester	PP13 second trimester
Early (*n* = 11)	0.93 ± 0.4	91 ± 11.6	60.2 ± 20.5	58.2 ± 270.9
Late (*n* = 89)	1.53 ± 0.6	96.68 ± 13.5	73.02 ± 19.8	276.08 ± 52.3
*P* value	0.005	0.186	0.047	0.761

**Table 4:** Validity of studied markers for preeclampsia in the first and second trimesters.

Marker	Cutoff	Sensitivity (95% CI)	Specificity % (95% CI)	Positive predictive value % (95% CI)	Negative predictive value % (95% CI)	Surface under curve (95% CI)
PAPPA first trimester	≤1.65	60.0 (49.7–69.7)	78.0 (68.6–85.7)	73.2 (62.2–82.4)	66.1 (56.8–74.6)	0.793 (0.733–0.854)
PP13 first trimester	≤88.5	77.0 (67.5–84.8)	82.0 (73.1–89.0)	81.1 (71.7–88.4)	78.1 (69.0–85.6)	0.879 (0.833–0.924)
PAPPA second trimester	≤100.5	71.0 (61.1 = 79.6)	71.0 (61.1 = 79.6)	71.0 (61.1 = 79.6)	71.0 (61.1 = 79.6)	0.809 (0.751–0.867)
PP13 second trimester	≤315.5	79.0 (69.7–86.5)	80.0 (70.5–87.3)	79.8 (70.5–87.2)	79.2 (70.0–86.6)	0.876 (0.829–0.924)

**Table 5:** Results of logistic regression analysis for two markers of preeclampsia in two time sessions, first and second trimesters.

Model	Constant	PAPPA coefficient (95% CI)	PP13 coefficient (95% CI)	Likelihood ratio PAPPA (95% CI)	Likelihood ratio PP13 (95% CI)	Chi-square (*P* value)
First trimester	10.958	−1.060 (−1.566–0.553)	−0.100 (−0.133–0.067)	0.347 (0.209–0.575)	0.905 (0.875–0.935)	136.1 (*P* < 0.001)
Second trimester	18.033	−0.0915 (−0.1277–0.0553)	−0.0262 (−0.0346–0.179)	0.913 (0.880–0.946)	0.974 (0.966–0.982)	138.9 (*P* < 0.001)

**Table 6:** Results of logistic regression analysis for two markers of preeclampsia in two time sessions, first and second trimesters (4 variables in sum).

Model	Constant	PAPPA coefficient (95% CI)	PP13 coefficient (95% CI)	PAPPA coefficient (95% CI)	PP13 coefficient (95% CI)	Chi-square (*P* value)
Four variables Simultaneously	24.534	−0.979 (−1.578–0.380)	−0.087 (−0.131–0.043)	−0.064 (−0.107–0.020)	−0.026 (−0.037–0.015)	178.04 (<0.0001)

**Table 7:** The validity factors of linear combinations of preeclampsia markers based on the three linear combinations.

Linear combination	Cutoff	Sensitivity (95% CI)	Specificity % (95% CI)	Positive predictive value % (95% CI)	Negative predictive value % (95% CI)	Surface under curve (95% CI)
PAPPA1 + (PP13_1/10)	≤10.9	86.0 (77.6–91.2)	80.0 (70.8–87.3)	81.1 (72.4–88.1)	85.1 (76.3–91.6)	0.919 (0.884–0.954)
PAPPA2 + (PP13_2/3)	≤214.5	89.0 (81.2–94.4)	81.0 (71.9–88.2)	82.4 (73.9–89.1)	88.0 (79.6–93.9)	0.932 (0.886–0.957)
(10 × PAPPA1) + PP13_1 + (PAPPA2/2) + (PP13_2/4)	≤238.5	91.0 (83.6–95.8)	91.0 (83.6–95.8)	91.0 (83.6–95.8)	91.0 (83.6–95.8)	0.968 (0.949–0.987)

**Table 8:** Validity factors in parallel tests of preeclampsia markers based on the first and second trimesters.

Time session	Positive result	Sensitivity (95% CI)	Specificity % (95% CI)	Positive predictive value % (95% CI)	Negative predictive value % (95% CI)
First trimester	Both positive	51.0 (40.8–61.1)	96.0 (90.1–98.9)	92.7 (82.4–98.0)	66.2 (57.8–73.8)
First trimester	At least one positive	86.0 (77.6–92.1)	64.0 (53.8–73.4)	70.5 (61.6–78.4)	82.1 (71.1–89.8)
Second trimester	Both positive	56.0 (45.7–65.9)	97.0 (91.5–99.4)	94.9 (85.9–98.9)	68.8 (60.5–76.3)
Second trimester	At least one positive	94.0 (87.4–97.8)	54.0 (43.7–74.8)	67.1 (58.7–74.8)	90.0 (79.5–96.2)

## References

[1] Heilmannl L, Seikmann U (1998). Hemodynamic and hemorhological profiles in women with proteinuric hypertension og pregnancy and in pregnant controls. *Archives of Gynecology and Obstetrics*.

[2] Chomian N (2003). Investigation of during of activity phase and method delivering in primipar with preeclampsia. *Raze Behzistan*.

[3] Chaftez I, kuhnreich I, Smmar M (2007). First trimester Placental protein 13 screening for preeclampsia and intra uterin growth restriction. *American Journal of Obstetrics and Gynecology*.

[4] Than NG, Pick E, Bellyei S (2004). Functional analyses of placental protein 13/galectin-13. *European Journal of Biochemistry*.

[5] Nicolaides KH, Bindra R, Turan OM (2006). A novel approach to first-trimester screening for early pre-eclampsia combining serum PP-13 and Doppler ultrasound. *Ultrasound in Obstetrics and Gynecology*.

[6] Romero R, Kusanovic JP, Than NG (2008). First-trimester maternal serum PP13 in the risk assessment for preeclampsia. *American Journal of Obstetrics and Gynecology*.

[7] Sekiya T, Kurahashi H, Udagaway Y (2008). Increased level of pregnancy assouiated protein A2 in the serum of preeclam patients. *Molecular Human Reproduction*.

[8] Hertig A, Berkane N, Lefevre G (2004). Maternal serum sFlt1 concentration is an early and reliable predictive marker of preeclampsia. *Clinical Chemistry*.

[9] Spencer K, Cowans NJ, Chefetz I, Tal J, Meiri H (2007). First-trimester maternal serum PP-13, PAPP-A and second-trimester uterine artery Doppler pulsatility index as markers of pre-eclampsia. *Ultrasound in Obstetrics and Gynecology*.

[10] Grill S, Rusterholz C, Zanetti-Dällenbach R (2009). Potential markers of preeclampsia—a review. *Reproductive Biology and Endocrinology*.

[11] Costa SL, Proctor L, Dodd JM (2008). Screening for placental insufficiency in high-risk pregnancies: is earlier better?. *Placenta*.

[12] Poon LCY, Maiz N, Valencia C, Plasencia W, Nicolaides KH (2009). First-trimester maternal serum pregnancy-associated plasma protein-A and pre-eclampsia. *Ultrasound in Obstetrics and Gynecology*.

[13] Audibert F, Boucoiran I, An N (2010). Screening for preeclampsia using first-trimester serum markers and uterine artery Doppler in nulliparous women. *American Journal of Obstetrics and Gynecology*.

[14] Bersinger NA, Smárason AK, Muttukrishna S, Groome NP, Redman CW (2003). Women with preeclampsia have increased serum levels of pregnancy-associated plasma protein A (PAPP-A), inhibin A, activin A, and soluble E-selectin. *Hypertension in Pregnancy*.

[15] Odibo AO, Zhong Y, Goetzinger KR (2011). First-trimester placental protein 13, PAPP-A, uterine artery Doppler and maternal characteristics in the prediction of pre-eclampsia. *Placenta*.

[16] Khalil A, Cowans NJ, Spencer K, Goichman S, Meiri H, Harrington K (2009). First trimester maternal serum placental protein 13 for the prediction of pre-eclampsia in women with a priori high risk. *Prenatal Diagnosis*.

[17] Burger O, Pick E, Zwickel J (2004). Placental protein 13 (PP-13): effects on cultured trophoblasts, and its detection in human body fluids in normal and pathological pregnancies. *Placenta*.

